# Construction of Pyroptosis-Related Prognostic and Immune Infiltration Signature in Bladder Cancer

**DOI:** 10.1155/2022/6429993

**Published:** 2022-12-14

**Authors:** Xiaoyi Du, Xin Zhao, Yu Tang, Wei Tang

**Affiliations:** ^1^Department of Urology, The First Affiliated Hospital of Chongqing Medical University, Chongqing, China; ^2^Department of Urology, The Affiliated Hospital of Southwest Medical University, Luzhou, Sichuan, China

## Abstract

Pyroptosis is a kind of programmed cell death related to inflammation, which is closely related to cancer. The goal of this study is to establish and verify pyroptosis-related gene signature to predict the prognosis of patients with bladder cancer (BLCA) and explore its relationship with immunity. Somatic mutation, copy number variation, correlation, and expression of 33 pyroptosis-related genes were evaluated based on The Cancer Genome Atlas (TCGA) database. BLCA cases were divided into two clusters by consistent clustering and found that pyroptosis-related genes were related to the overall survival (OS) of BLCA. The least absolute shrinkage and selection operator (LASSO) Cox regression was used to construct the signature (including 7 pyroptosis-realated genes). Survival analysis curve and receiver operating characteristic curve (ROC) showed that this signature could predict the prognosis of BLCA patients. Univariate and multivariate Cox regression analysis showed the independent prognostic value of this model. Immune infiltration analysis showed that the six types of immune cells have significantly different infiltrations. The effect of immunotherapy is better in the low-risk group. In summary, our effort indicated the potential role of pyroptosis-related genes in BLCA and provided new perspectives on the prognosis of BLCA and new ideas for immunotherapy.

## 1. Introduction

Bladder cancer (BLCA) usually refers to the tumor originating from the bladder epithelium, and its incidence rate is increasing continuously since 1990 [1]. BLCA is one of the most common malignant tumor in the urinary system [2, 3]. The pathological types of BLCA are mainly divided into three types: urothelial carcinoma, squamous cell carcinoma, and adenocarcinoma; among them, urothelial carcinoma accounts for more than 90% of total bladder cancer [4]. Then, according to the degree of tumor invasion, BLCA can be divided into nonmuscle invasive bladder cancer (NMIBC) and muscle invasive bladder cancer (MIBC) [5]. In recent years, the understanding of the pathogenesis of BLCA has increased a lot; the treatment methods include surgery, radiotherapy, chemotherapy, immunotherapy, etc., but the recurrence rate and mortality of patients are still high, and the recurrence rate of NMIBC is as high as 70%; the 5-year survival rate of MIBC is less than 50% [6]. Improving the overall survival rate of patients and reducing the recurrence rate remain a major clinical challenge. Therefore, it is important to determine new effective treatment targets, judge the prognosis of bladder cancer patients in a multidimensional model, further understand the pathogenesis of BLCA, and formulate more effective comprehensive treatment strategies.

In 1992, pyroptosis was first observed in Shigella infected macrophages, at that time, it was defined as apoptosis because they had the same characteristics, such as DNA fragmentation, nuclear concentration, and caspase dependence [7]. It was not until 2001 that researchers further clarified that macrophage death caused by bacterial infection is a death mode completely different from apoptosis and named it caspase-1-dependent programmed necrosis [8]. In 2018, the nomenclature committee on cell death (NCCD) proposed to define pyroptosis as a regulated cell death (RCD) [9]. At present, it is found that it mainly relies on gasdermin protein family members to form plasma membrane pores, usually, but not always as a consequence of inflammatory caspase activation; it can lead to the continuous expansion of cells until the cell membrane breaks and the release of cell contents, which triggers a strong inflammatory response; thus, initially, pyroptosis was considered to be a key mechanism to fight infection [10–12]. Now, more and more studies have shown that the key components of pyroptosis, inflammatory vesicles, gasdermin protein, and proinflammatory cytokines are related to tumor occurrence, invasion, and metastasis [13–15]. It has been reported that pyroptosis-related genes are significantly upregulated in tumor tissue samples and urine of bladder cancer patients, and different pyroptosis-related inflammasomes may affect the pathological characteristics of bladder cancer [16–18]. However, the relationship between pyroptosis and BLCA has yet to be investigated fully.

In this study, we used the public database The Cancer Genome Atlas (TCGA) to obtain data on patients with bladder cancer. The differences in the expression of pyroptosis-related genes in normal bladder tissues and BLCA tissues were analyzed. Consensus Clustering and LASSO Cox regression were used to establish subgroups; then, the relationship between pyroptosis-related genes and the prognosis, immune infiltration, and immunotherapy of patients with BLCA was explored. Compared with traditional clinical prediction, we can establish a pyroptosis-related gene signature to more accurately predict the prognosis of BLCA patients and provide some clinical guidance for individualized immunotherapy.

## 2. Materials and Methods

### 2.1. Datasets

The flowchart of this study is shown in Figure S1. We obtained the RNA sequence (RNA-seq) data of patients with bladder cancer from the TCGA database (https://portal.gdc.cancer.gov/repository) as well as the corresponding clinical and pathological information. For external validation datasets (GSE32548 and GSE31684) were obtained from GEO (http://www.ncbi.nlm.nih.gov/geo/). Patients without survival information were excluded. Based on the previously published literature [19–26], we finally selected a total of 33 genes (AIM2, CASP1, CASP3, CASP4, CASP5, CASP6, CASP8, CASP9, ELANE, GPX4, GSDMA, GSDMB, GSDMC, GSDMD, GSDME, IL18, IL1B, IL6, NLRC4, NLRP1, NLRP2, NLRP3, NLRP6, NLRP7, NOD1, NOD2, PJVK, PLCG1, PRKACA, PYCARD, SCAF11, TIRAP, and TNF) that strongly associated with cell pyroptosis as pyroptosis-related genes. Patients in the immunotherapy data were from The Cancer Immunome Atlas (TCIA) (https://tcia.at/). The maftools Bioconductor package was used to read the MAF files to count the variants in each sample and visualize it. The limma Bioconductor package was used to identify differentially expressed genes between normal and tumor tissues.

### 2.2. Consensus Clustering

In the first, we applied the “survival” R package to perform univariate Cox regression analysis on pyroptosis-related genes and initially screened out prognostic-related genes that were significantly associated with overall survival (OS). The ConsensuClusterPlus R package was used to perform consensus clustering of each BLCA sample based on prognostic-related gens expression data. The Kaplan-Meier method was used for OS analysis.

### 2.3. Establishment of Pyroptosis-Related Gene Prognostic Signature and Independent Prognostic Analysis

Next, patients with TCGA data set were randomly divided into train cohort and test cohort. Based on the prognostic-related genes that were initially screened, we performed LASSO Cox regression analysis on the train cohort to establish the best prognostic signature. We could get the optimal lambda value and a list of prognostic genes with coefficients generated by the LASSO model. The calculation of the risk score is based on the following formula:

(1) $$\begin{array}{c} \text{Risk score}=\overset{\text{k}}{\underset{\text{i}}{\sum }}\text{x}\text{i}*\text{yi} \end{array}$$

The *k*, xi, and yi represented the number of signature genes, the gene expression level, and the coefficient index, respectively. In the train cohort, the BLCA patients were divided into high-risk and low-risk groups according to the median risk score. The difference in survival between the high-risk group and low-risk group was analyzed by the Kaplan-Meier survival curves. ROC analysis was used to further evaluate the prognostic ability of gene signature. In addition, in the test cohort and entire cohort, the same formula and statistical methods were used to verify the predictive power of gene signature. Univariate and multivariate Cox regression models were used to analyze the gene signature and clinicopathological information. Then, the nomogram is drawn for clinical practice. CIBERSORT algorithm was used to analyze the relationship between prognositc signature and immune cell. We used TCIA database to generate an immunophenoscore (IPS) for each sample to compare the immunotherapy responses of different risk groups.

### 2.4. Cell Culture

The BLCA cell lines (5637 and T24) were obtained from the American Type Culture Collection (Rockville, MD, USA). Human urothelial cells were obtained from the ScienCell (Carlsbad, CA). T24 and 5637 were cultured in Roswell Park Memorial Institute (RPMI)-1640 medium (Gibco, CA, USA), and HUC was cultured in F12 Nutrient Mixture (GIBCO, Grand Island, NY); both supplemented with 10% fetal bovine Serum (fetal bovine serum, Pan Biotech, Germany) and 1% penicillin/streptomycin (In Vitromycin, Carlsbad, USA). The cells were incubated at 37°C in 5% CO2.

### 2.5. Total RNA Extraction and Quantitative Real-Time PCR (qPCR)

Total RNA from cells was extracted by the Steady Pure Quick RNA Extraction Kit (AG21023, Accurate Biotechnology, Hunan, China) and reverse transcribed (Takara, Dalian, China) to acquire cDNAs. qPCR was performed on the BIO-RAD PCR system using BlazeTaq™ SYBR Green qPCRMix (Genomics, Guangzhou, China). The internal control for this qPCR was human *β*-actin. The relative expression levels of these genes were calculated using the 2^−ΔΔCt^ method. Primers for AIM2, CASP6,GSDMB, and GSDMD can be found in Table S1.

### 2.6. Statistical Analysis

The Wilcox test was used to compare the expression levels of genes between normal bladder and BLCA tissues. The Kaplan-Meier analysis with a two-sided log-rank test was used to compare OS of patients in different groups. ROC curves and the area under curves (AUC) were applied to evaluate the prediction accuracy of the signature. Univariate and multivariate Cox regression analyses were conducted to identify the independent predictor for OS. All statistical analyses were performed with R software (v4.0.5), GraphPad Prism 5 (GraphPad), and Excel (Excel 2007®); the *P* values < 0.05 were considered statistically significant.

## 3. Results

### 3.1. Overview of Genetic Changes of Pyroptosis-Related Genes in BLCA

**Figure 1:**
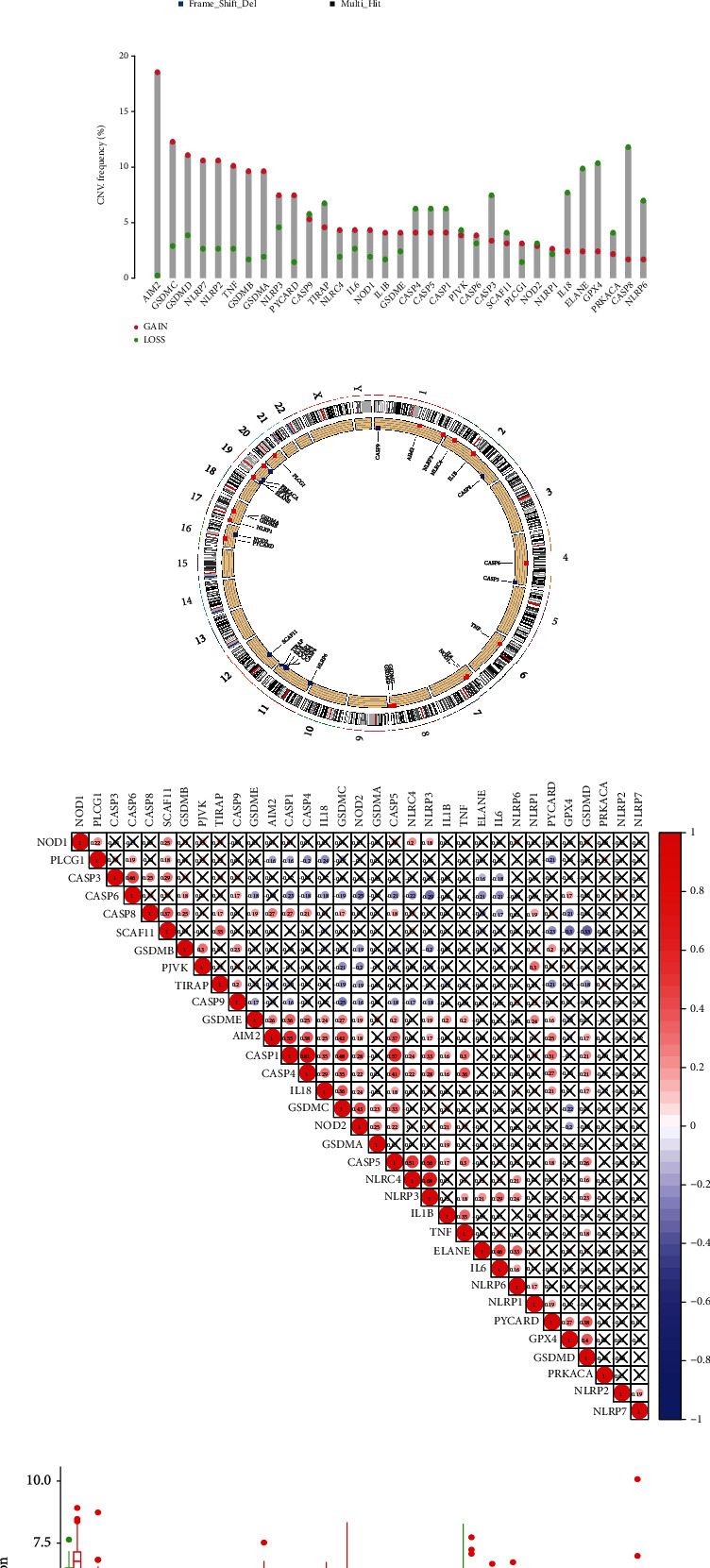
The characteristics, correlations, and differences of pyroptosis-related genes in BLCA. (a) The mutation frequency of 33 pyroptosis-related genes in 412 BLCA patients from TCGA-STAD cohort. The upper barplot showed tumor mutation burden. The right number indicated the mutation frequency in each gene. The corresponding colors are annotated at the bottom to indicate different mutation types. (b) The CNV variation frequency of pyroptosis-related genes in TCGA cohort. The height of the column represented the alteration frequency. (c) The location of CNV alteration of pyroptosis-related genes on 23 chromosomes using TCGA cohort. (d) Correlation analysis of pyroptosis-related genes in BLCA. (e) The expressions of pyroptosis-related genes between normal tissues and BLCA tissues. The asterisks represented the statistical *P* value ( ^∗^P < 0.05;  ^∗∗^P < 0.01;  ^∗∗∗^P < 0.001).

We first summarized the incidence of copy number variation and somatic mutations of 33 pyroptosis-related genes in bladder cancer. Among the 412 TCGA samples, 95 samples (approximately 23.06%) had mutations in pyroptosis-related genes. The results showed that the mutation frequency of SCAF11, NLRP7, and NLRP2 was 3%; the mutation frequency of CASP8, CASP1, NLRP3, PLCG1, and CASP5 was 2%; the mutation frequency of NLRP1, NOD2, NLRP6, NLRC4, GSDMD, ELANE, CASP9, NOD1, and GSDMC was 1%, and the other 16 pyroptosis-related genes were not found in any mutations in BLCA samples (Figure 1(a)). As shown in Figure 1(b), copy number variations (CNV) were common in pyroptosis-related genes. The location of CNV alteration of pyroptosis-related genes on chromosomes was shown in Figure 1(c). The correlation between pyroptosis-related genes was shown in Figure 1(d). The difference in mRNA expression levels of pyroptosis-related genes between normal and BLCA samples was shown in Figure 1(e).

### 3.2. Tumor Classification Based on Differential Expression of Pyroptosis-Related Genes

**Figure 2:**
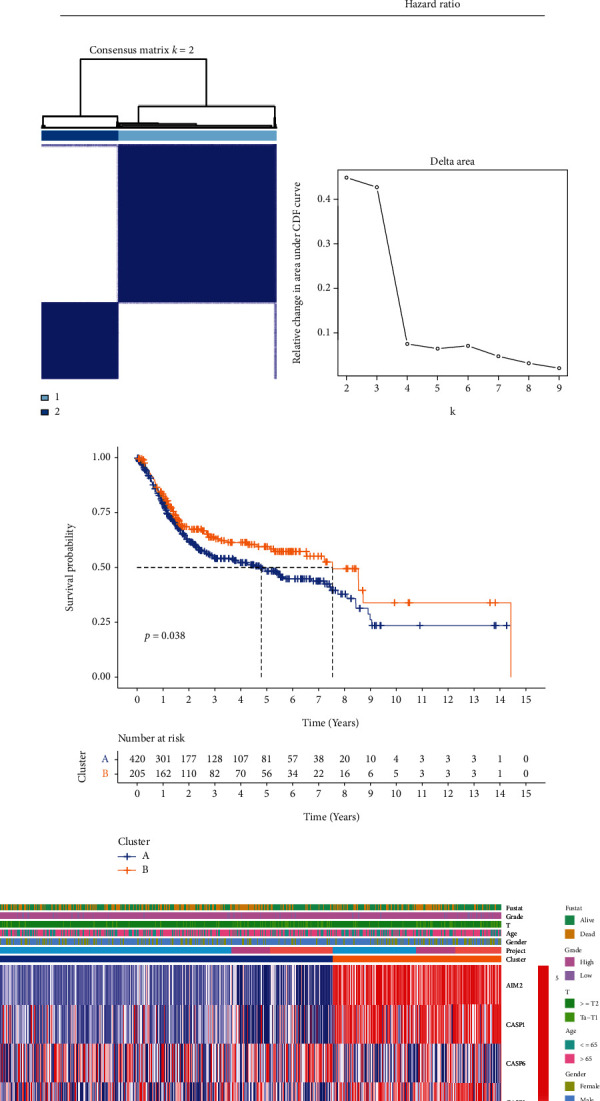
Two subgroups were divided according to the Consensus Clustering to compare the survival differences. (a) The forest showed the hazard ratio (95% CI) and *P* value of selected pyroptosis-related genes by univariate Cox regression. (b) Consensus clustering matrix for *k* = 2. (c) CDF curves for *k* = 2 − 9. (d) The Kaplan-Meier curves of overall survival (OS) for BLCA in two clusters. (e) Heatmap and the clinicopathologic characters of the two clusters classified by 8 genes.

Univariate Cox regression analysis was used to initially screen survival-related genes. The 8 genes (CASP8, GSDMB, AIM2, CASP1, GSDMD, CASP6, PRKACA, and CASP9) meeting the *P* < 0.05 criteria were retained for further analysis (Figure 2(a)). Consensus cluster analysis was used to explore the relationship between the expression of 8 prognostic-related regulators and BLCA subtypes. By increase the clustering variable (*k*) from 2 to 9, when *k* = 2, there was a high intragroup correlation and a low intergroup correlation, indicating that BLCA patients with TCGA, GSE31684, and GSE32548 could be suitably divided into two clusters based on 8 prognostic-related genes (Figures 2(b) and 2(c)). The overall survival rate of the two groups was compared, and there was a difference in OS between the two clusters (Figure 2(d)). Then, the heatmap showed the association between cluster, 8-gene expression, clinical information, and pathological characteristics (Figure 2(e)).

### 3.3. Construction and Validation of Prognostic Signature for Pyroptosis-Related Regulators

**Figure 3:**
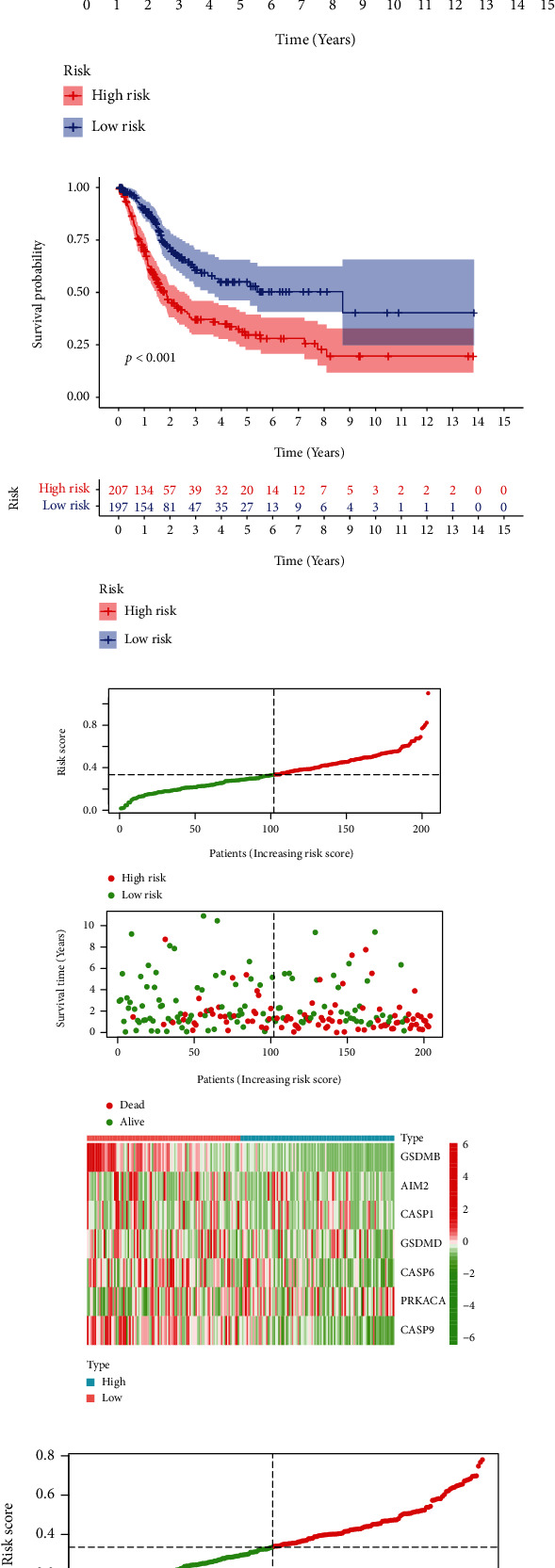
Prognostic value of pyroptosis-related genes in BLCA patients. (a, b) Least absolute shrinkage and selection operator (LASSO) Cox regression identified a risk prognosis model. (c) Coefficients of 7 pyroptosis-relate genes. (d–f) In the train cohort (d), test cohort (e), and entire cohort (f), the Kaplan-Meier curves suggested that the low-risk group had better OS than the high-risk group. (g–i) The risk scores distribution, BLCA patients' survival status, and expression heatmap in the train cohort (g), test cohort (h), and entire cohort (i). (j–l) The train cohort (j), test cohort (k), and entire cohort (l): receiver operating characteristic (ROC) curves of pyroptosis-relate genes for predicting the 1-, 3-, 5-year survival of BLCA patients.

Patients from TCGA were randomly divided into train cohort and test cohort. In the train cohort, the eight genes (CASP8, GSDMB, AIM2, CASP1, GSDMD, CASP6, PRKACA, and CASP9) were performed with the LASSO Cox regression analysis; then, a prognostic signature with 7 genes (AIM2, CASP1, CASP6, CASP9, GSDMB, GSDMD, and PRKACA) was constructed, and the corresponding coefficients were obtained (Figures 3(a)–3(c)). Based on the median risk score, patients in the train cohort were divided into high-risk and low-risk groups. In order to verify this prognostic signature, we introduced this risk model into the test cohort and the entire cohort. According to the median risk value of the train cohort, patients in the test cohort and the entire cohort were divided into high-risk and low-risk groups. The Kaplan-Meier survival curves of OS showed that patients in the high-risk group tended to have a shorter OS than the low-risk group (Figures 3(d)–3(f)). The risk score and survival status of each case were sorted and displayed on the dot chart. We could found a significant difference in OS between the high-risk and the low-risk groups. The heatmap showed the 7 genes expression differences between the high-risk and low-risk groups (Figures 3(g)–3(i)). By using GSVA enrichment analysis, the biological characteristics of patients in high- and low-risk groups were compared, and it was discovered that the high-risk group had an enrichment of cancer-related pathways (Figure S2a). The time-dependent ROC curve was applied to evaluate the predictive effect of the prognostic signature; the AUC values were as follows, 0.704 (train cohort, 1 year), 0.712 (test cohort, 1 year), 0.707 (entire cohort, 1 year), 0.727 (train cohort, 3 years), 0.655 (test cohort, 3 years), 0.696 (entire cohort, 3 years), 0.745 (train cohort, 5 years), 0.666 (test cohort, 5 years), and 0.708 (entire cohort, 5 years) (Figures 3(j)–3(l)).

We integrated datasets GSE32584 and GSE31684 as external validation. Patients were divided into high-risk and low-risk groups using the risk model obtained from the train cohort. It was observed that patients in the low-risk group had a better prognosis than the high-risk group (Figures S2b–2d). The 1-, 3-, and 5-year ROC curves showed that the risk model still had high AUC values (Figure S2e). It is suggested that our risk model has good ability in predicting the prognosis of BLCA patients. We selected 4 differentially expressed genes for qPCR validation in normal urothelial cell line and bladder cancer cell lines, and the results were as predicted by bioinformatics (Figure S2f).

### 3.4. Independent Prognostic Value of the Prognostic Signature

**Figure 4:**
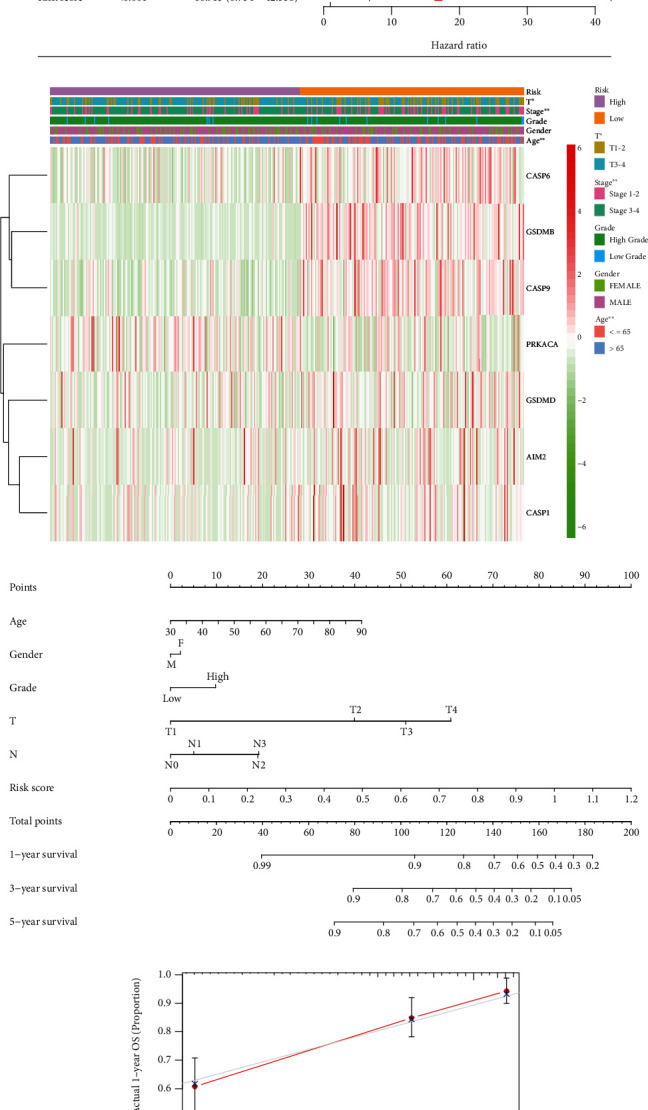
The risk model is an independent prognostic factor and nomogram for predicting the survival probability of patients with BLCA. (a) Univariate and (b) multivariate Cox regression analyses. (c) Heatmap and clinicopathologic features of high-risk and low-risk groups. The asterisks represented the statistical *P* value (  ^∗^*P* < 0.05; ^∗∗^*P* < 0.01; ^∗∗∗^*P* < 0.001). (d) Prognostic nomogram for BLCA patients. (e–g) Calibration curves for the nomogram at (e) 1-, (f) 3-, and (g) 5-year.

Then, univariate and multivariate Cox regression analysis were applied to assess whether the risk score derived from the 7-gene signature model could performed as an independent prognostic factor in the entire cohort (Figures 4(a) and 4(b)). The results indicated that this model could be used as an independent prognostic indicator. In addition, we used heatmaps to show the relationship between these 7 genes and clinical features. Six genes (CASP6, GSDMB, CASP9, GSDMD, AIM2, and CASP1) were upregulated, while PRKACA genes were downregulated in the low-risk group. It was also found that the patient's age, T, and Stage were statistically different between the low-risk group and the high-risk group (*P* < 0.05) (Figure 4(c)). Based on the risk scores model, a nomogram containing 5 clinical features is constructed. Clinicians can easily combine common clinical features with our model to predict the survival expectations of patients with BLCA (Figure 4(d)). The calibration chart shows that the nomogram predictions, and actual observations are in good agreement in the 1-year, 3-year, and 5-year survival rates (Figures 4(e)–4(g)).

### 3.5. Identification of the Relationship between the Prognostic Signature and Immune

**Figure 5:**
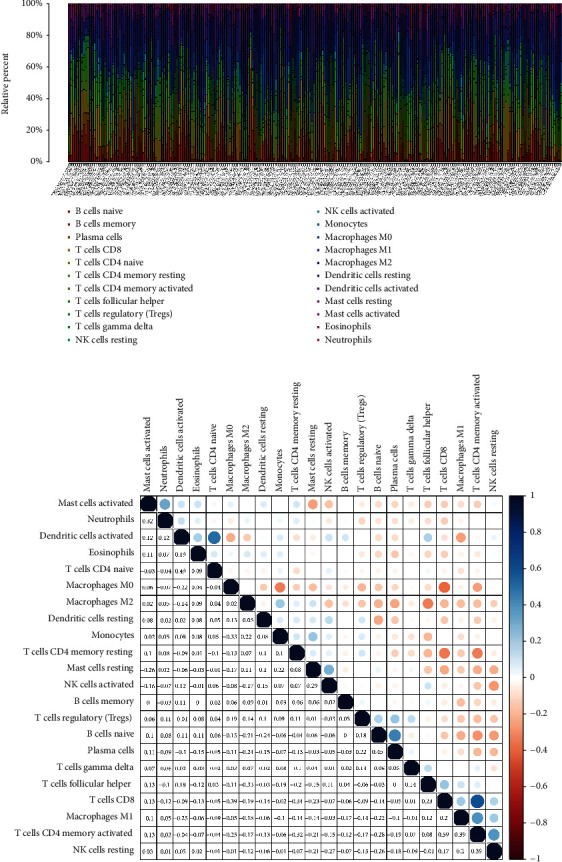
Tumor-infiltrating immune cells. (a) Barplot displays the ratio of 22 types of immune cells in BLCA samples. Column names: sample ID. (b) Correlation analysis for the 22 types of immune cells.

In order to better study the interaction between the prognostic signature and the immune microenvironment, the CIBERSORT algorithm was used to detect the ratio of tumor-infiltrating immune cells in BLCA. Firstly, the relative content distribution of 22 types of tumor-infiltrating immune cells (TICs) in each sample and the correlation between 22 TICs were evaluated (Figures 5(a) and 5(b)).

**Figure 6:**
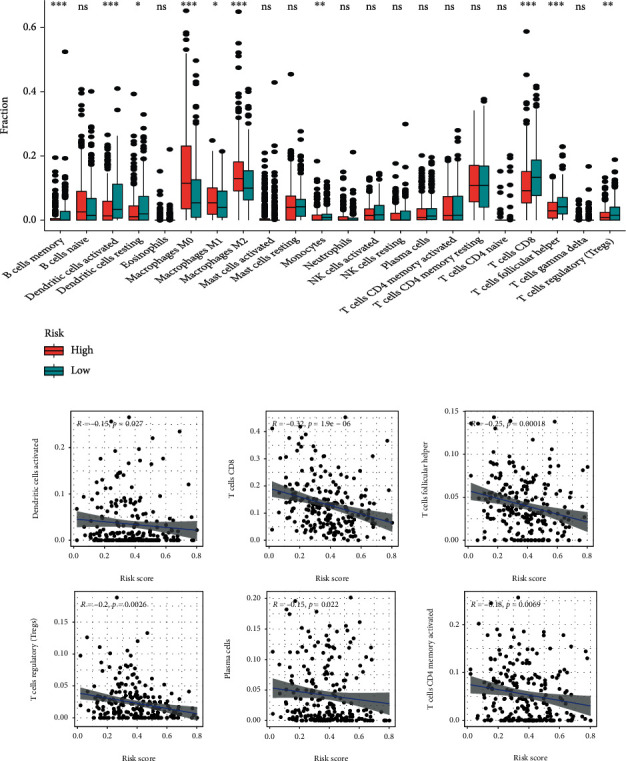
Correlation analysis between risk score and tumor-infiltrating immune cells. (a) The infiltrating levels of 22 immune cell types in high- and low-risk group. (b) Relationships between the risk score and infiltration abundances of immune cell types.

**Figure 7:**
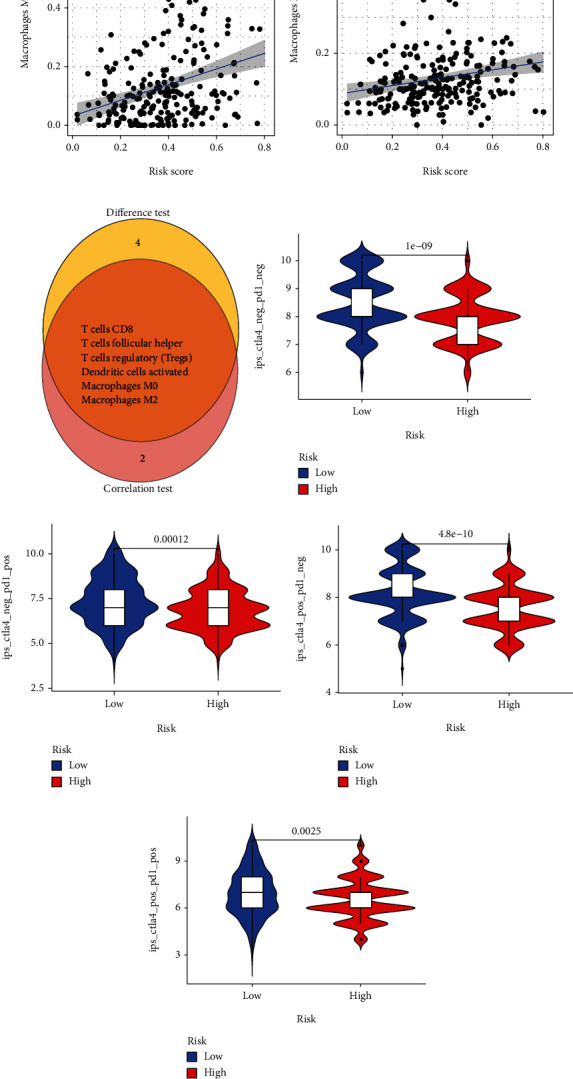
Correlation analysis between risk score and tumor-infiltrating immune cells. Prediction of immunotherapy response. (a, b) Relationships between the risk score and infiltration abundances of immune cell types. (c) Venn plot displays six types of immune cells shared by difference and correlation tests. (d–g) Comparison of IPS between low-risk group and high-risk group.

The overlapping results of difference analysis (Figure 6(a)) and correlation analysis (Figure 6(b) and Figures 7(a) and 7(b)) could found that 6 TICs were potentially correlated with prognostic signature (Figure 7(c)). Activated Dendritic cells, CD8+T cells, follicular helper T cells, and regulatory T (Treg) cells were found to have negative correlations with the prognostic signature, M0 Macrophages, and M2 Macrophages showed positive correlations with it.

Immunotherapy is one of the main methods of tumor treatment. The number and activation status of TICs are closely related to the immunotherapy and prognosis of many tumors [27–30]. Immunophenoscore (IPS) is considered to be an effective predictor of immunotherapy, so we studied the relationship between IPS and risk scores. We found that the low-risk group had higher IPS than the high-risk group. These results indicated that the low-risk group was treated with anti-PD1 alone and anti-ctla4 alone, or the combination of the two has a higher positive response rate (Figures 7(d)–7(g)).

## 4. Discussion

BLCA is the eleventh most common malignant tumor in the world. Its morbidity and mortality are high, and it brings a significant health burden to the society. Immunotherapy brings new opportunities for patients with BLCA, but due to the low response to immunotherapy, only a few patients can benefit from it. Therefore, it is necessary to further discover effective biological prediction models in clinical practice. Pyroptosis is a new type of programmed cell death that causes inflammation and cell lysis [31, 32]. Pyroptosis is related to many diseases and is considered a double-edged sword in tumors, especially its dual role in tumor formation and tumor microenvironment. Existing studies have shown that pyroptosis can inhibit the growth of gastric cancer and colon cancer, but it can promote cervical cancer. However, the prognostic value and mechanism of pyroptosis-related genes in BLCA remain to be studied.

In this study, we systematically explored the expression, gene mutations, and clinical significance of 33 currently known pyroptosis-related genes in BLCA. The results showed that there were differences in the expression of many pyroptosis-related genes in BLCA and adjacent normal tissues. SCAF11 (3%), NLRP7 (3%), NLRP2 (3%), CASP5 (2%), PLCG1 (2%), NLRP3 (2%), CASP1 (2%), CASP8 (2%), GSDMC (1%), NOD1 (1%), CASP9 (1%), ELANE (1%), GSDMD (1%), NLRC4 (1%), NLRP6 (1%), NOD2 (1%), and NLRP1 (1%) occurred somatic mutations as well as most of them had high frequencies of CNV in BLCA. Because of the bidirectional role of pyroptosis in tumors, a single pyroptosis-related gene seems to be unreliable for the diagnosis and prognosis of BLCA. This inspired us to use multiple pyroptosis genes to explore the diagnostic and prognostic value of pyroptosis. Two clusters produced by the consensus clustering analysis based on the differentially expressed genes showed significant differences in the OS. In order to further evaluate the prognostic value of these pyroptosis-related genes, LASSO Cox regression analysis was used to establish a risk model. A new pyroptosis-related gene signature containing 7 genes (AIM2, CASP1, CASP6, CASP9, GSDMB, GSDMD, and PRKACA) was constructed, and verified. AUC results showed that the signature has good accuracy in predicting the survival of BLCA patients. Univariate and multivariate analysis suggested that the model is an independent risk factor that independently affects the prognosis of BLCA.

Pyroptosis is a form of RCD; when cells are stimulated, aspartate-specific cysteine-containing proteases (Caspase) can be activated by the intracellular inflammasome, which cleaves Gasdermin (GSDM) protein family to form the plasma membrane pores, causing inflammatory cell death, which is of great significance in cancer and cancer treatment [9]. GSDMD and GSDMB are members of the GSDM gene family, and both have an N-terminal domain, which can bind to phospholipids on the cell membrane, open the channel for the release of inflammatory factors, and mediate the occurrence of pyroptosis [21, 33]. In related studies of tumor cells, GSDMB was found to be highly expressed in breast, gastric, liver, cervical, and colon cancers; therefore, some scholars believe that GSDMB may be involved in cancer progression and metastasis as an oncogene [34, 35]. However, recently, Zhou et al. found that in a mouse colon tumor model, granzyme A secreted by toxic lymphocytes can directly cleave and activate GSDMB, induce pyroptosis of target cells and increase the immune clearance rate of tumors [36]. There are also many studies on GSDMD in tumor treatment, and it is often regarded as a tumor suppressor gene that plays a role in tumor cells. When Wang et al. explored the relationship between gastric cancer cells and GSDMD, they found that high expression of GSDMD could inhibit the proliferation of gastric cancer cells, and activation of GSDMD-induced pyroptosis could promote tumor cell death and exert the anticancer properties of GSDMD [37]. In addition, AIM2, as an inflammasome participating in the classical pyroptosis pathway, can play a key role in the occurrence and development of cell pyroptosis by cleaving GSDMD and releasing IL-1*β* and IL-18 [38]. Studies have shown that AIM2 is related to the occurrence and development of various tumors such as colon cancer, prostate cancer, and breast cancer [39–41]. In conclusion, GSDMD, GSDMB, and AIM2 are all promising new targets in cancer therapy, which may be involved in the death mechanism of tumor cell therapy through pyroptosis. Except for pyroptosis, there are also abnormal proliferation, abnormal differentiation, and abnormal apoptosis involved in the process of tumorigenesis. Caspases are a class of proteolytic enzymes that mediate apoptosis and expressed in human tissue cells and tumor cells, which are extremely important apoptosis initiating and executing factors as well as involved in the process of pyroptosis [42]. Among them, CASP6 and CASP9 mainly mediate the apoptotic process, and CASP1 mainly induces pyroptosis, but there are also interactions among the three, and CASP6 is reported to regulate the activation of CASP1 to promote the formation of GSDMD-induced pyroptosis [43]. PRKACA plays an important role in the development and progression of many cancers [44, 45]. However, the molecular mechanism of PRKACA involved in the occurrence and development of bladder cancer is still unclear and needs to be investigated and explored in depth. Therefore, we believe that these seven genes may be involved in the occurrence and development of bladder cancer by regulating pyroptosis or apoptosis. Existing studies have proved that it is not difficult to see that pyroptosis has a complex role in cancer. Thus, when evaluating the prognosis of patients, the impact of a certain gene of pyroptosis on patients cannot be discussed separately, whereas it should be considered comprehensively. Hence, the most direct and effective way is to establish a prognosis-related model.

Immune cell infiltration in tumor tissue plays an important role in cancer cell proliferation, invasion, migration, etc. Dendritic cells (DC) can initiate and regulate the adaptive immune response, which is the basis for the antitumor immune response [46]. The number of CD8+T cells has implication for chemotherapy, and immunotherapy could improve the therapeutic effects of tumor treatment; it has been confirmed in patients with gastric cancer, breast cancer, and melanoma [47–49]. In a mouse model of breast cancer with high mutation burden, follicular helper T cells can mediate the response to checkpoint inhibitors and enhance the antitumor response [50]. Treg cells can suppress tumor immunity and are related to tumor aggressiveness [51, 52]. M2 macrophages have anti-inflammatory effects, and polarized M2 macrophages are called tumor-associated macrophages (TAMs) that can promote tumor growth and invasion [53]. Immunotherapy has a great role in the personalized treatment of cancers such as the urinary system. Induction of pyroptosis is considered to be a new and potential cancer treatment measure. Studies have found that induction of pyroptosis combined with immunotherapy can enhance anticancer activity [54]. By analyzing the proportion and types of immune cells, we found that there are significant differences in immune cells between the high- and low-risk groups, so we further analyzed the correlation between the risk score and immune cells. It was found that activated Dendritic cells, CD8+T cells, follicular helper T cells, and regulatory T (Treg) cells were higher in the low-risk group, and they were negatively correlated with the risk score. The M0 Macrophages and M2 Macrophages were higher in the high-risk group; they are positively correlated with risk scores. Unexpectedly, in our study, the proportion of regulatory T (Treg) cells in the low-risk group was higher than in the high-risk group. Therefore, how Treg cells play a role in patients with bladder cancer and the relationship with pyroptosis need further research. We believe that one possible reason for this difference is that regulatory T (Treg) cells could regulate the overactive inflammatory response caused by pyroptosis in the tumor microenvironment. Finally, we explored the predictive ability of risk signatures and immunotherapy response and found that patients in the low-risk group responded better to immunotherapy. In summary, the prognostic model based on pyroptosis-related regulators may provide new insights for the prognosis of immunotherapy for bladder cancer and help clinical medical decision making.

## 5. Conclusions

In our study, it was found that there are differences in the expression of pyroptosis-related regulators in normal bladder tissue and bladder cancer, and the OS of patients grouped according to the expression of pyroptosis-related regulators is different. A prognostic signature of 7 genes has been established and verified that it is an independent prognostic factor for patients with bladder cancer and is related to the tumor immune microenvironment. Of course, our research has limitations; further multicenter studies and experimental investigations are needed. However, our research provides a new genetic marker for predicting the prognosis of patients with BLCA and provides an important basis for further research on the relationship between pyroptosis-related regulators and bladder cancer immunity.

## Data Availability

The data set analyzed in this study can be found in TCGA and GEO database.

## Supplementary Materials

Supplementary Materials Figure S1: study flowchart. Figure S2: validation of risk score. (a) Heatmap visualization of GSVA analysis results for different risk groups, with red and blue representing activated and inhibited biological pathways, respectively. (b) The Kaplan-Meier analysis of patients with high and low risk scores. (c) Risk scores distribution and patient survival status. (d) Heatmap showing the expression profile of five risk model genes. (e) ROC curves and AUC for the accuracy of predicting 1-, 3-, and 5-year survival based on risk model. (f) Expression levels of 4 risk model genes in bladder cancer cell line and normal urothelial cell line by RT-PCR. Table S1: primers used for RT-qPCR.
