# Supplementary material for: Construction of Pyroptosis-Related Prognostic and Immune Infiltration Signature in Bladder Cancer
Source: Dis Markers. 2022 Dec 14;2022:6429993. doi: 10.1155/2022/6429993 (PMC9771655; doi:10.1155/2022/6429993)
Supplement: Supplementary Materials — Figure S1: study flowchart. Figure S2: validation of risk score. (a) Heatmap visualization of GSVA analysis results for different risk groups, with red and blue representing activated and inhibited biological pathways, respectively. (b) The Kaplan-Meier analysis of patients with high and low risk scores. (c) Risk scores distribution and patient survival status. (d) Heatmap showing the expression profile of five risk model genes. (e) ROC curves and AUC for the accuracy of predicting 1-, 3-, and 5-year survival based on risk model. (f) Expression levels of 4 risk model genes in bladder cancer cell line and normal urothelial cell line by RT-PCR. Table S1: primers used for RT-qPCR. [file 6429993.f1.docx]

Supplementary Material

# Supplementary Figures and Tables

## Supplementary Figures


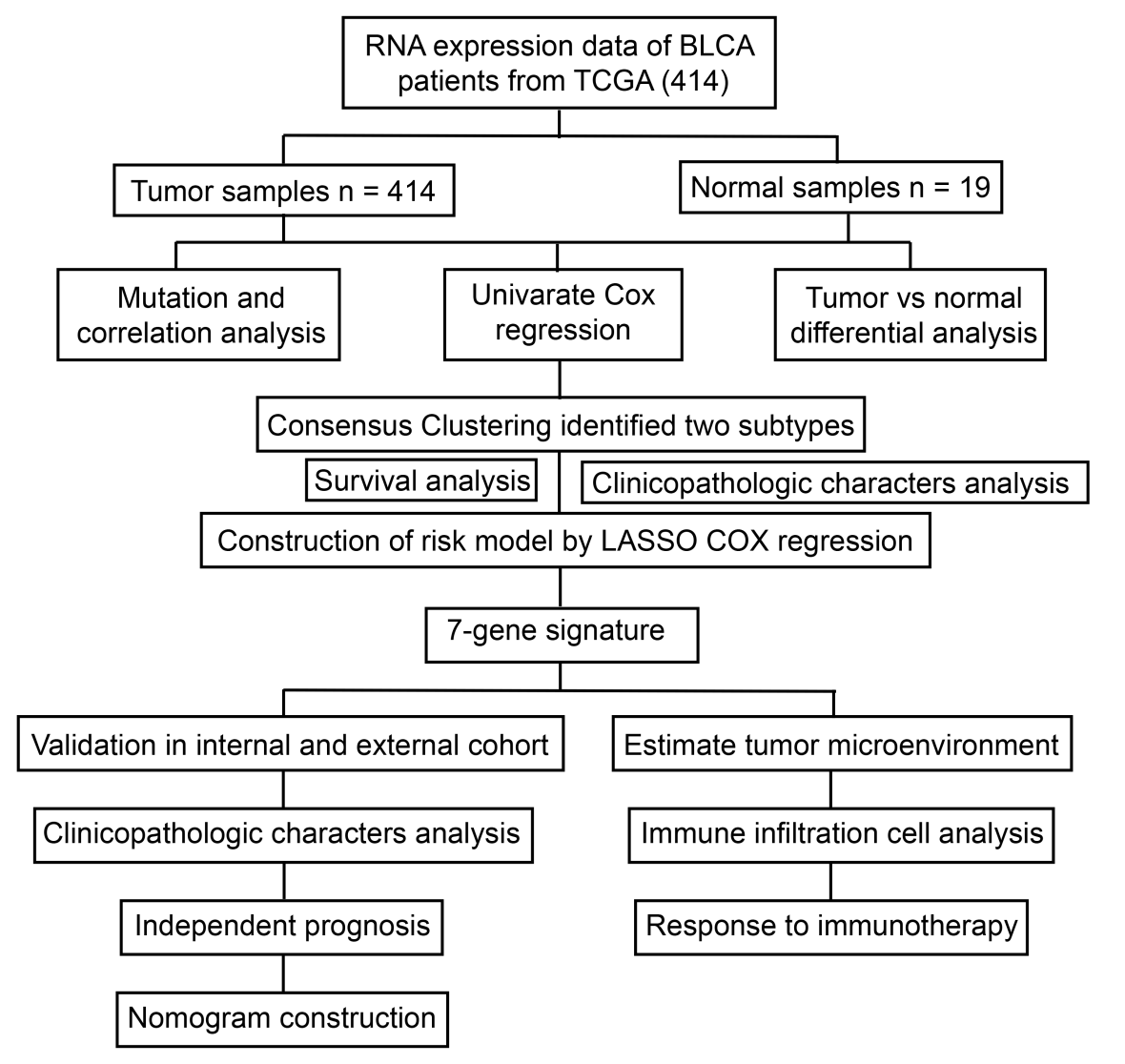


**Figure S1.** Study flowchart.


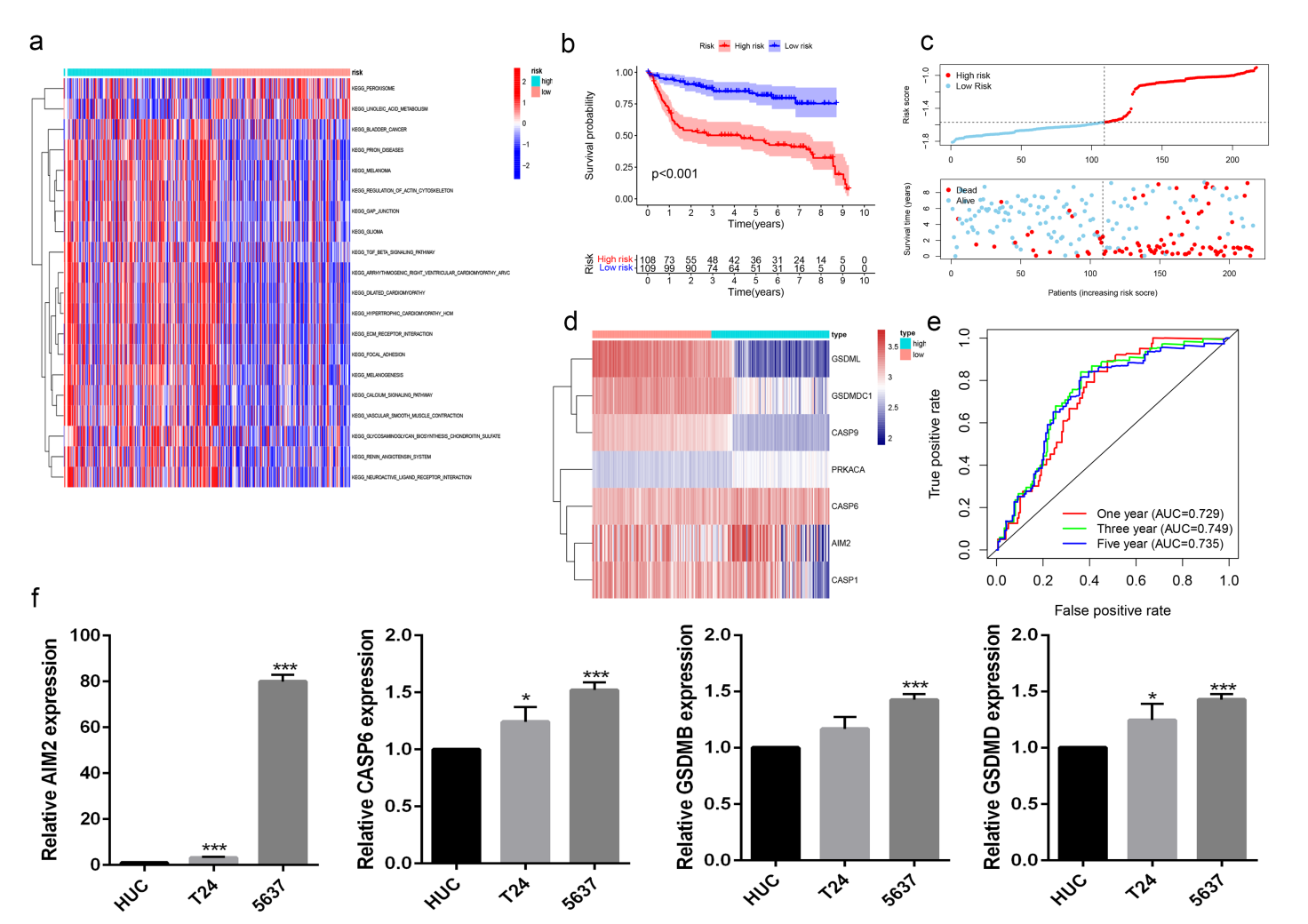


**Figure S2.** Validation of risk score. (a) Heatmap visualization of GSVA analysis results for different risk groups, with red and blue representing activated and inhibited biological pathways, respectively. (b) Kaplan-Meier analysis of patients with high and low risk scores. (c) Risk scores distribution and patient survival status. (d) heatmap showing the expression profile of five risk model genes. (e) ROC curves and AUC for the accuracy of predicting 1-, 3-, and 5-year survival based on risk model. (f) Expression levels of 4 risk model genes in bladder cancer cell line and normal urothelial cell line by RT-PCR.

## Supplementary Tables

**Table S1. Primers used for RT-qPCR**

| Genes | Forward (5’- to 3’-) | Reverse (5’- to 3’-) |  |  |
| --- | --- | --- | --- | --- |
| AIM2 | AAATGATGTCGCAAAGCAACG | ACCATAACTGGCAAACAGCG |  |  |
| CASP6 | TGCCGATTGCTTTGTGTGTG | GTGCTGGTTTCCCCGACAT |  |  |
| GSDMB | GAGGTCAGAGAGGAGTTGGTC | AGCAGCGGAATCTATCAGCA |  |  |
| GSDMD | TGGTTAGGAAGCCCTCAAGC | CTGCCCTGTATCTGCCCATC |  |  |
| β-actin | ACCCCGTGCTGCTGACCGAG | TCCCGGCCAGCCAGGTCCA |  |  |
